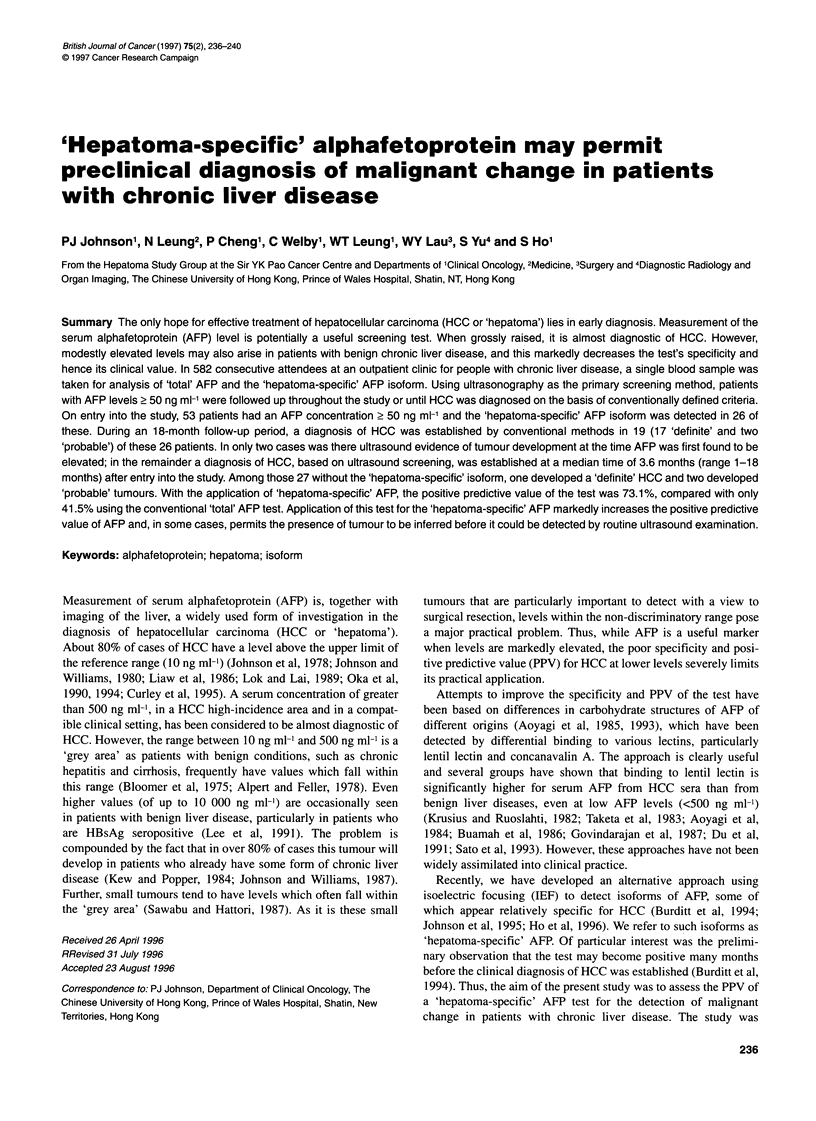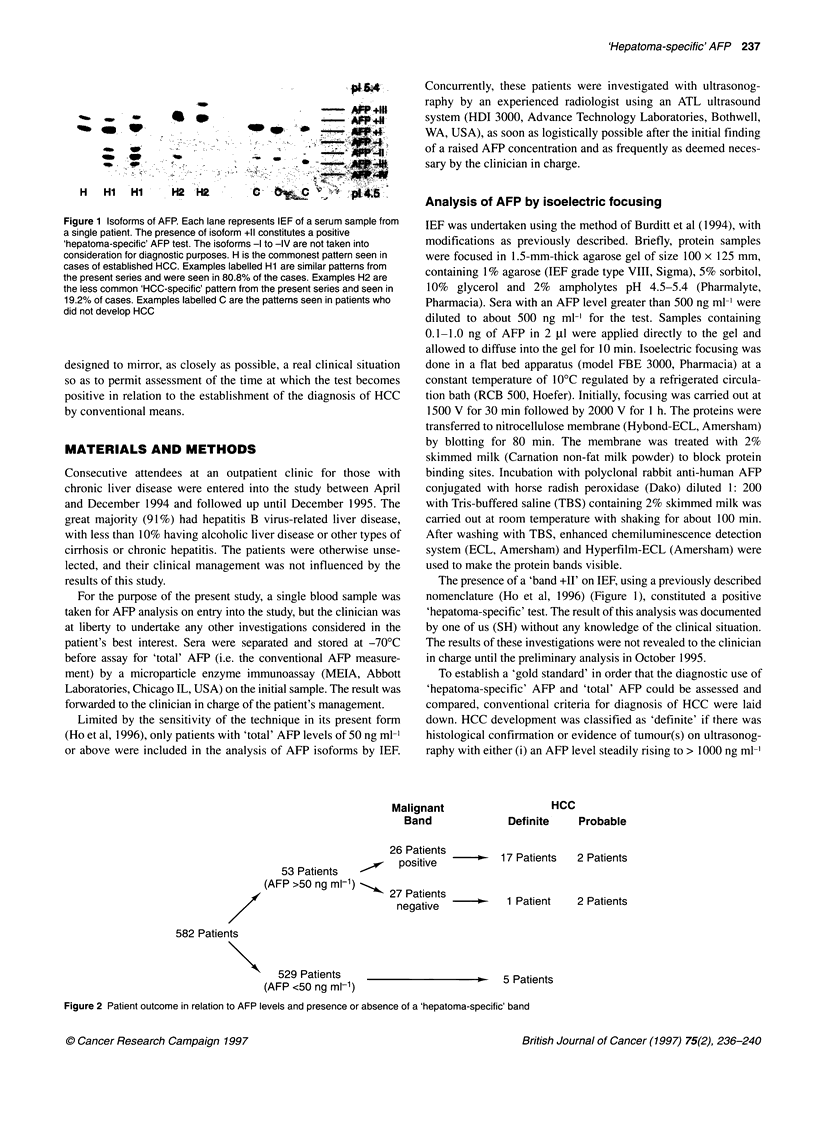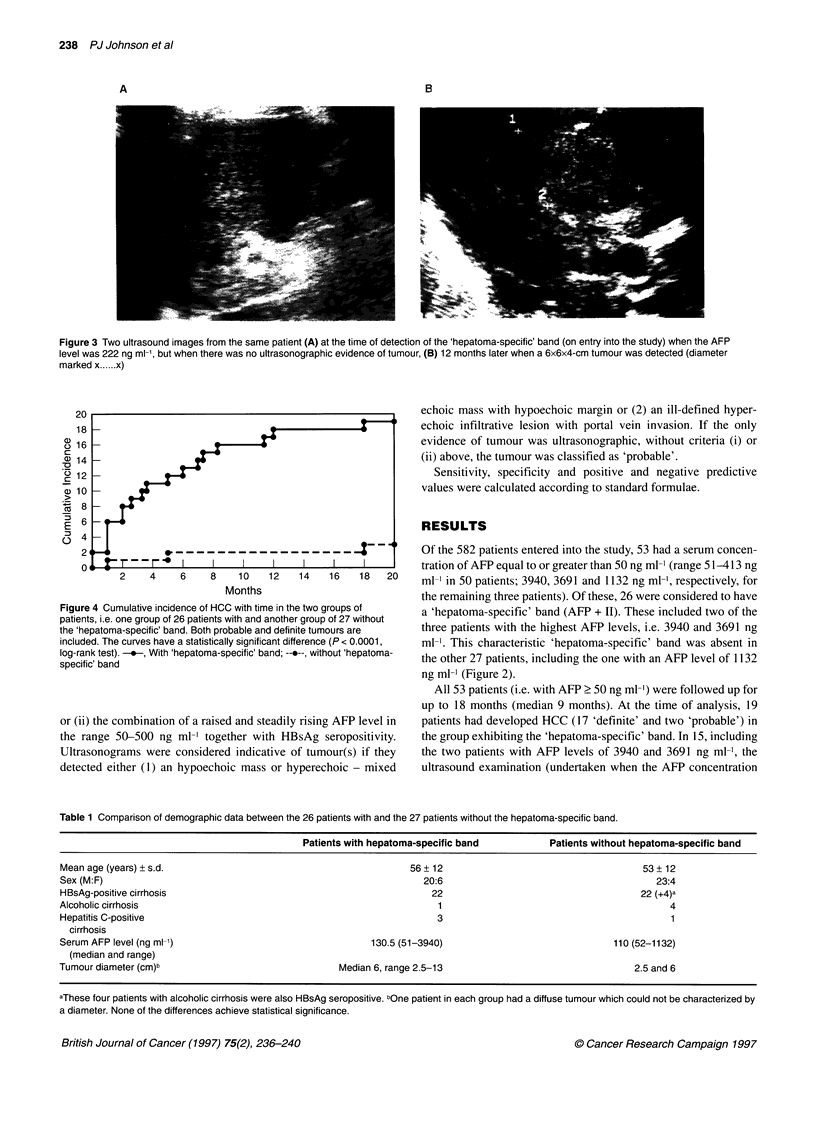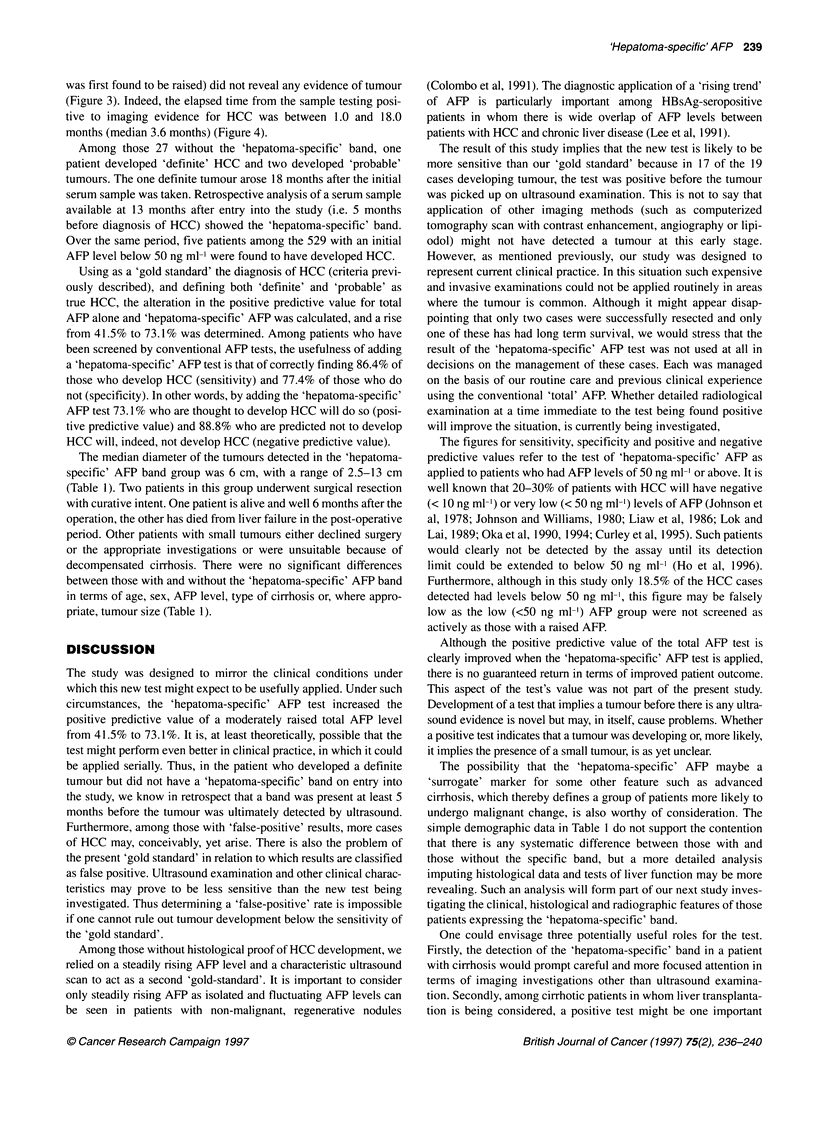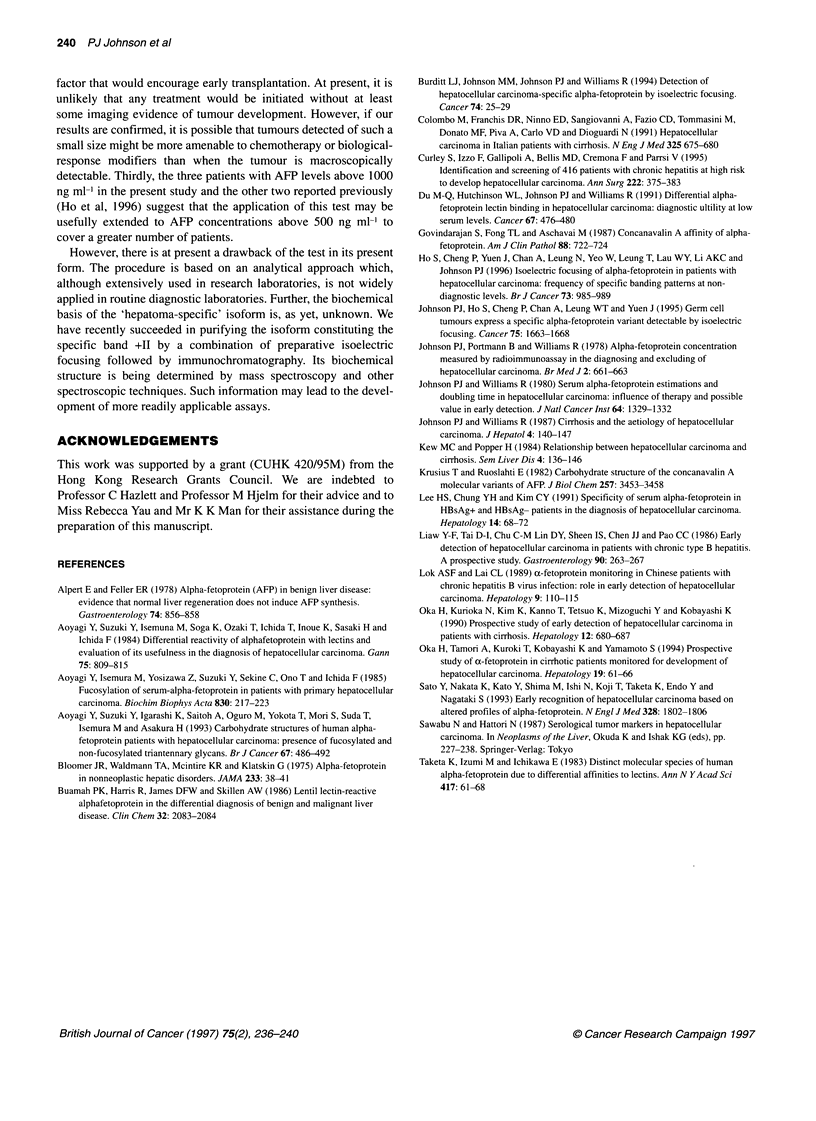# 'Hepatoma-specific' alphafetoprotein may permit preclinical diagnosis of malignant change in patients with chronic liver disease.

**DOI:** 10.1038/bjc.1997.39

**Published:** 1997

**Authors:** P. J. Johnson, N. Leung, P. Cheng, C. Welby, W. T. Leung, W. Y. Lau, S. Yu, S. Ho

**Affiliations:** Hepatoma Study Group, Sir YK Pao Cancer Centre, Shatin, NT, Hong Kong.

## Abstract

**Images:**


British Journal of Cancer (1997) 75(2), 236–240
© 1997 Cancer Research Campaign

### 'Hepatoma-specific' alphafetoprotein may permit preclinical diagnosis of malignant change in patients with chronic liver disease

**PJ Johnson[1], N Leung[2], P Cheng[1], C Welby[1], WT Leung[1], WY Lau[3], S Yu[4] and S Ho[1]**

From the Hepatoma Study Group at the Sir YK Pao Cancer Centre and Departments of [1]Clinical Oncology, [2]Medicine, [3]Surgery and [4]Diagnostic Radiology and Organ Imaging, The Chinese University of Hong Kong, Prince of Wales Hospital, Shatin, NT, Hong Kong

**Summary** The only hope for effective treatment of hepatocellular carcinoma (HCC or 'hepatoma') lies in early diagnosis. Measurement of the serum alphafetoprotein (AFP) level is potentially a useful screening test. When grossly raised, it is almost diagnostic of HCC. However, modestly elevated levels may also arise in patients with benign chronic liver disease, and this markedly decreases the test's specificity and hence its clinical value. In 582 consecutive attendees at an outpatient clinic for people with chronic liver disease, a single blood sample was taken for analysis of 'total' AFP and the 'hepatoma-specific' AFP isoform. Using ultrasonography as the primary screening method, patients with AFP levels ≥ 50 ng $ml^{-1}$ were followed up throughout the study or until HCC was diagnosed on the basis of conventionally defined criteria. On entry into the study, 53 patients had an AFP concentration ≥ 50 ng $ml^{-1}$ and the 'hepatoma-specific' AFP isoform was detected in 26 of these. During an 18-month follow-up period, a diagnosis of HCC was established by conventional methods in 19 (17 'definite' and two 'probable') of these 26 patients. In only two cases was there ultrasound evidence of tumour development at the time AFP was first found to be elevated; in the remainder a diagnosis of HCC, based on ultrasound screening, was established at a median time of 3.6 months (range 1–18 months) after entry into the study. Among those 27 without the 'hepatoma-specific' isoform, one developed a 'definite' HCC and two developed 'probable' tumours. With the application of 'hepatoma-specific' AFP, the positive predictive value of the test was 73.1%, compared with only 41.5% using the conventional 'total' AFP test. Application of this test for the 'hepatoma-specific' AFP markedly increases the positive predictive value of AFP and, in some cases, permits the presence of tumour to be inferred before it could be detected by routine ultrasound examination.

**Keywords:** alphafetoprotein; hepatoma; isoform

Measurement of serum alphafetoprotein (AFP) is, together with imaging of the liver, a widely used form of investigation in the diagnosis of hepatocellular carcinoma (HCC or 'hepatoma'). About 80% of cases of HCC have a level above the upper limit of the reference range (10 ng $ml^{-1}$) (Johnson et al, 1978; Johnson and Williams, 1980; Liaw et al, 1986; Lok and Lai, 1989; Oka et al, 1990, 1994; Curley et al, 1995). A serum concentration of greater than 500 ng $ml^{-1}$, in a HCC high-incidence area and in a compatible clinical setting, has been considered to be almost diagnostic of HCC. However, the range between 10 ng $ml^{-1}$ and 500 ng $ml^{-1}$ is a 'grey area' as patients with benign conditions, such as chronic hepatitis and cirrhosis, frequently have values which fall within this range (Bloomer et al, 1975; Alpert and Feller, 1978). Even higher values (of up to 10 000 ng $ml^{-1}$) are occasionally seen in patients with benign liver disease, particularly in patients who are HBsAg seropositive (Lee et al, 1991). The problem is compounded by the fact that in over 80% of cases this tumour will develop in patients who already have some form of chronic liver disease (Kew and Popper, 1984; Johnson and Williams, 1987). Further, small tumours tend to have levels which often fall within the 'grey area' (Sawabu and Hattori, 1987). As it is these small tumours that are particularly important to detect with a view to surgical resection, levels within the non-discriminatory range pose a major practical problem. Thus, while AFP is a useful marker when levels are markedly elevated, the poor specificity and positive predictive value (PPV) for HCC at lower levels severely limits its practical application.

Attempts to improve the specificity and PPV of the test have been based on differences in carbohydrate structures of AFP of different origins (Aoyagi et al, 1985, 1993), which have been detected by differential binding to various lectins, particularly lentil lectin and concanavalin A. The approach is clearly useful and several groups have shown that binding to lentil lectin is significantly higher for serum AFP from HCC sera than from benign liver diseases, even at low AFP levels (<500 ng $ml^{-1}$) (Krusius and Ruoslahti, 1982; Taketa et al, 1983; Aoyagi et al, 1984; Buamah et al, 1986; Govindarajan et al, 1987; Du et al, 1991; Sato et al, 1993). However, these approaches have not been widely assimilated into clinical practice.

Recently, we have developed an alternative approach using isoelectric focusing (IEF) to detect isoforms of AFP, some of which appear relatively specific for HCC (Burditt et al, 1994; Johnson et al, 1995; Ho et al, 1996). We refer to such isoforms as 'hepatoma-specific' AFP. Of particular interest was the preliminary observation that the test may become positive many months before the clinical diagnosis of HCC was established (Burditt et al, 1994). Thus, the aim of the present study was to assess the PPV of a 'hepatoma-specific' AFP test for the detection of malignant change in patients with chronic liver disease. The study was

*Received 26 April 1996*
*RRevised 31 July 1996*
*Accepted 23 August 1996*

*Correspondence to:* PJ Johnson, Department of Clinical Oncology, The Chinese University of Hong Kong, Prince of Wales Hospital, Shatin, New Territories, Hong Kong

**Figure 1** Isoforms of AFP. Each lane represents IEF of a serum sample from a single patient. The presence of isoform +II constitutes a positive 'hepatoma-specific' AFP test. The isoforms –I to –IV are not taken into consideration for diagnostic purposes. H is the commonest pattern seen in cases of established HCC. Examples labelled H1 are similar patterns from the present series and were seen in 80.8% of the cases. Examples H2 are the less common 'HCC-specific' pattern from the present series and seen in 19.2% of cases. Examples labelled C are the patterns seen in patients who did not develop HCC

designed to mirror, as closely as possible, a real clinical situation so as to permit assessment of the time at which the test becomes positive in relation to the establishment of the diagnosis of HCC by conventional means.

### MATERIALS AND METHODS

Consecutive attendees at an outpatient clinic for those with chronic liver disease were entered into the study between April and December 1994 and followed up until December 1995. The great majority (91%) had hepatitis B virus-related liver disease, with less than 10% having alcoholic liver disease or other types of cirrhosis or chronic hepatitis. The patients were otherwise unselected, and their clinical management was not influenced by the results of this study.

For the purpose of the present study, a single blood sample was taken for AFP analysis on entry into the study, but the clinician was at liberty to undertake any other investigations considered in the patient's best interest. Sera were separated and stored at –70°C before assay for 'total' AFP (i.e. the conventional AFP measurement) by a microparticle enzyme immunoassay (MEIA, Abbott Laboratories, Chicago IL, USA) on the initial sample. The result was forwarded to the clinician in charge of the patient's management.

Limited by the sensitivity of the technique in its present form (Ho et al, 1996), only patients with 'total' AFP levels of 50 ng $ml^{-1}$ or above were included in the analysis of AFP isoforms by IEF. Concurrently, these patients were investigated with ultrasonography by an experienced radiologist using an ATL ultrasound system (HDI 3000, Advance Technology Laboratories, Bothwell, WA, USA), as soon as logistically possible after the initial finding of a raised AFP concentration and as frequently as deemed necessary by the clinician in charge.

### Analysis of AFP by isoelectric focusing

IEF was undertaken using the method of Burditt et al (1994), with modifications as previously described. Briefly, protein samples were focused in 1.5-mm-thick agarose gel of size 100 × 125 mm, containing 1% agarose (IEF grade type VIII, Sigma), 5% sorbitol, 10% glycerol and 2% ampholytes pH 4.5–5.4 (Pharmalyte, Pharmacia). Sera with an AFP level greater than 500 ng $ml^{-1}$ were diluted to about 500 ng $ml^{-1}$ for the test. Samples containing 0.1–1.0 ng of AFP in 2 μl were applied directly to the gel and allowed to diffuse into the gel for 10 min. Isoelectric focusing was done in a flat bed apparatus (model FBE 3000, Pharmacia) at a constant temperature of 10°C regulated by a refrigerated circulation bath (RCB 500, Hoefer). Initially, focusing was carried out at 1500 V for 30 min followed by 2000 V for 1 h. The proteins were transferred to nitrocellulose membrane (Hybond-ECL, Amersham) by blotting for 80 min. The membrane was treated with 2% skimmed milk (Carnation non-fat milk powder) to block protein binding sites. Incubation with polyclonal rabbit anti-human AFP conjugated with horse radish peroxidase (Dako) diluted 1: 200 with Tris-buffered saline (TBS) containing 2% skimmed milk was carried out at room temperature with shaking for about 100 min. After washing with TBS, enhanced chemiluminescence detection system (ECL, Amersham) and Hyperfilm-ECL (Amersham) were used to make the protein bands visible.

The presence of a 'band +II' on IEF, using a previously described nomenclature (Ho et al, 1996) (Figure 1), constituted a positive 'hepatoma-specific' test. The result of this analysis was documented by one of us (SH) without any knowledge of the clinical situation. The results of these investigations were not revealed to the clinician in charge until the preliminary analysis in October 1995.

To establish a 'gold standard' in order that the diagnostic use of 'hepatoma-specific' AFP and 'total' AFP could be assessed and compared, conventional criteria for diagnosis of HCC were laid down. HCC development was classified as 'definite' if there was histological confirmation or evidence of tumour(s) on ultrasonography with either (i) an AFP level steadily rising to > 1000 ng $ml^{-1}$

**Figure 2** Patient outcome in relation to AFP levels and presence or absence of a 'hepatoma-specific' band

© Cancer Research Campaign 1997

**Figure 3** Two ultrasound images from the same patient **(A)** at the time of detection of the 'hepatoma-specific' band (on entry into the study) when the AFP level was 222 ng ml$^{-1}$, but when there was no ultrasonographic evidence of tumour, **(B)** 12 months later when a 6x6x4-cm tumour was detected (diameter marked x......x)

**Figure 4** Cumulative incidence of HCC with time in the two groups of patients, i.e. one group of 26 patients with and another group of 27 without the 'hepatoma-specific' band. Both probable and definite tumours are included. The curves have a statistically significant difference ($P < 0.0001$, log-rank test). —●—, With 'hepatoma-specific' band; --●--, without 'hepatoma-specific' band or (ii) the combination of a raised and steadily rising AFP level in the range 50–500 ng ml$^{-1}$ together with HBsAg seropositivity. Ultrasonograms were considered indicative of tumour(s) if they detected either (1) an hypoechoic mass or hyperechoic – mixed echoic mass with hypoechoic margin or (2) an ill-defined hyperechoic infiltrative lesion with portal vein invasion. If the only evidence of tumour was ultrasonographic, without criteria (i) or (ii) above, the tumour was classified as 'probable'.

Sensitivity, specificity and positive and negative predictive values were calculated according to standard formulae.

### RESULTS

Of the 582 patients entered into the study, 53 had a serum concentration of AFP equal to or greater than 50 ng ml$^{-1}$ (range 51–413 ng ml$^{-1}$ in 50 patients; 3940, 3691 and 1132 ng ml$^{-1}$, respectively, for the remaining three patients). Of these, 26 were considered to have a 'hepatoma-specific' band (AFP + II). These included two of the three patients with the highest AFP levels, i.e. 3940 and 3691 ng ml$^{-1}$. This characteristic 'hepatoma-specific' band was absent in the other 27 patients, including the one with an AFP level of 1132 ng ml$^{-1}$ (Figure 2).

All 53 patients (i.e. with AFP $\geq$ 50 ng ml$^{-1}$) were followed up for up to 18 months (median 9 months). At the time of analysis, 19 patients had developed HCC (17 'definite' and two 'probable') in the group exhibiting the 'hepatoma-specific' band. In 15, including the two patients with AFP levels of 3940 and 3691 ng ml$^{-1}$, the ultrasound examination (undertaken when the AFP concentration

**Table 1** Comparison of demographic data between the 26 patients with and the 27 patients without the hepatoma-specific band.

| | Patients with hepatoma-specific band | Patients without hepatoma-specific band |
|---|---|---|
| Mean age (years) ± s.d. | 56 ± 12 | 53 ± 12 |
| Sex (M:F) | 20:6 | 23:4 |
| HBsAg-positive cirrhosis | 22 | 22 (+4)[a] |
| Alcoholic cirrhosis | 1 | 4 |
| Hepatitis C-positive cirrhosis | 3 | 1 |
| Serum AFP level (ng ml$^{-1}$) (median and range) | 130.5 (51–3940) | 110 (52–1132) |
| Tumour diameter (cm)[b] | Median 6, range 2.5–13 | 2.5 and 6 |

[a]These four patients with alcoholic cirrhosis were also HBsAg seropositive. [b]One patient in each group had a diffuse tumour which could not be characterized by a diameter. None of the differences achieve statistical significance.

© Cancer Research Campaign 1997

was first found to be raised) did not reveal any evidence of tumour (Figure 3). Indeed, the elapsed time from the sample testing positive to imaging evidence for HCC was between 1.0 and 18.0 months (median 3.6 months) (Figure 4).

Among those 27 without the 'hepatoma-specific' band, one patient developed 'definite' HCC and two developed 'probable' tumours. The one definite tumour arose 18 months after the initial serum sample was taken. Retrospective analysis of a serum sample available at 13 months after entry into the study (i.e. 5 months before diagnosis of HCC) showed the 'hepatoma-specific' band. Over the same period, five patients among the 529 with an initial AFP level below 50 ng $ml^{-1}$ were found to have developed HCC.

Using as a 'gold standard' the diagnosis of HCC (criteria previously described), and defining both 'definite' and 'probable' as true HCC, the alteration in the positive predictive value for total AFP alone and 'hepatoma-specific' AFP was calculated, and a rise from 41.5% to 73.1% was determined. Among patients who have been screened by conventional AFP tests, the usefulness of adding a 'hepatoma-specific' AFP test is that of correctly finding 86.4% of those who develop HCC (sensitivity) and 77.4% of those who do not (specificity). In other words, by adding the 'hepatoma-specific' AFP test 73.1% who are thought to develop HCC will do so (positive predictive value) and 88.8% who are predicted not to develop HCC will, indeed, not develop HCC (negative predictive value).

The median diameter of the tumours detected in the 'hepatoma-specific' AFP band group was 6 cm, with a range of 2.5–13 cm (Table 1). Two patients in this group underwent surgical resection with curative intent. One patient is alive and well 6 months after the operation, the other has died from liver failure in the post-operative period. Other patients with small tumours either declined surgery or the appropriate investigations or were unsuitable because of decompensated cirrhosis. There were no significant differences between those with and without the 'hepatoma-specific' AFP band in terms of age, sex, AFP level, type of cirrhosis or, where appropriate, tumour size (Table 1).

### DISCUSSION

The study was designed to mirror the clinical conditions under which this new test might expect to be usefully applied. Under such circumstances, the 'hepatoma-specific' AFP test increased the positive predictive value of a moderately raised total AFP level from 41.5% to 73.1%. It is, at least theoretically, possible that the test might perform even better in clinical practice, in which it could be applied serially. Thus, in the patient who developed a definite tumour but did not have a 'hepatoma-specific' band on entry into the study, we know in retrospect that a band was present at least 5 months before the tumour was ultimately detected by ultrasound. Furthermore, among those with 'false-positive' results, more cases of HCC may, conceivably, yet arise. There is also the problem of the present 'gold standard' in relation to which results are classified as false positive. Ultrasound examination and other clinical characteristics may prove to be less sensitive than the new test being investigated. Thus determining a 'false-positive' rate is impossible if one cannot rule out tumour development below the sensitivity of the 'gold standard'.

Among those without histological proof of HCC development, we relied on a steadily rising AFP level and a characteristic ultrasound scan to act as a second 'gold-standard'. It is important to consider only steadily rising AFP as isolated and fluctuating AFP levels can be seen in patients with non-malignant, regenerative nodules (Colombo et al, 1991). The diagnostic application of a 'rising trend' of AFP is particularly important among HBsAg-seropositive patients in whom there is wide overlap of AFP levels between patients with HCC and chronic liver disease (Lee et al, 1991).

The result of this study implies that the new test is likely to be more sensitive than our 'gold standard' because in 17 of the 19 cases developing tumour, the test was positive before the tumour was picked up on ultrasound examination. This is not to say that application of other imaging methods (such as computerized tomography scan with contrast enhancement, angiography or lipiodol) might not have detected a tumour at this early stage. However, as mentioned previously, our study was designed to represent current clinical practice. In this situation such expensive and invasive examinations could not be applied routinely in areas where the tumour is common. Although it might appear disappointing that only two cases were successfully resected and only one of these has had long term survival, we would stress that the result of the 'hepatoma-specific' AFP test was not used at all in decisions on the management of these cases. Each was managed on the basis of our routine care and previous clinical experience using the conventional 'total' AFP. Whether detailed radiological examination at a time immediate to the test being found positive will improve the situation, is currently being investigated,

The figures for sensitivity, specificity and positive and negative predictive values refer to the test of 'hepatoma-specific' AFP as applied to patients who had AFP levels of 50 ng $ml^{-1}$ or above. It is well known that 20–30% of patients with HCC will have negative (< 10 ng $ml^{-1}$) or very low (< 50 ng $ml^{-1}$) levels of AFP (Johnson et al, 1978; Johnson and Williams, 1980; Liaw et al, 1986; Lok and Lai, 1989; Oka et al, 1990, 1994; Curley et al, 1995). Such patients would clearly not be detected by the assay until its detection limit could be extended to below 50 ng $ml^{-1}$ (Ho et al, 1996). Furthermore, although in this study only 18.5% of the HCC cases detected had levels below 50 ng $ml^{-1}$, this figure may be falsely low as the low (<50 ng $ml^{-1}$) AFP group were not screened as actively as those with a raised AFP.

Although the positive predictive value of the total AFP test is clearly improved when the 'hepatoma-specific' AFP test is applied, there is no guaranteed return in terms of improved patient outcome. This aspect of the test's value was not part of the present study. Development of a test that implies a tumour before there is any ultrasound evidence is novel but may, in itself, cause problems. Whether a positive test indicates that a tumour was developing or, more likely, it implies the presence of a small tumour, is as yet unclear.

The possibility that the 'hepatoma-specific' AFP maybe a 'surrogate' marker for some other feature such as advanced cirrhosis, which thereby defines a group of patients more likely to undergo malignant change, is also worthy of consideration. The simple demographic data in Table 1 do not support the contention that there is any systematic difference between those with and those without the specific band, but a more detailed analysis imputing histological data and tests of liver function may be more revealing. Such an analysis will form part of our next study investigating the clinical, histological and radiographic features of those patients expressing the 'hepatoma-specific' band.

One could envisage three potentially useful roles for the test. Firstly, the detection of the 'hepatoma-specific' band in a patient with cirrhosis would prompt careful and more focused attention in terms of imaging investigations other than ultrasound examination. Secondly, among cirrhotic patients in whom liver transplantation is being considered, a positive test might be one important

© Cancer Research Campaign 1997

factor that would encourage early transplantation. At present, it is unlikely that any treatment would be initiated without at least some imaging evidence of tumour development. However, if our results are confirmed, it is possible that tumours detected of such a small size might be more amenable to chemotherapy or biological-response modifiers than when the tumour is macroscopically detectable. Thirdly, the three patients with AFP levels above 1000 ng $ml^{-1}$ in the present study and the other two reported previously (Ho et al, 1996) suggest that the application of this test may be usefully extended to AFP concentrations above 500 ng $ml^{-1}$ to cover a greater number of patients.

However, there is at present a drawback of the test in its present form. The procedure is based on an analytical approach which, although extensively used in research laboratories, is not widely applied in routine diagnostic laboratories. Further, the biochemical basis of the 'hepatoma-specific' isoform is, as yet, unknown. We have recently succeeded in purifying the isoform constituting the specific band +II by a combination of preparative isoelectric focusing followed by immunochromatography. Its biochemical structure is being determined by mass spectroscopy and other spectroscopic techniques. Such information may lead to the development of more readily applicable assays.

### ACKNOWLEDGEMENTS

This work was supported by a grant (CUHK 420/95M) from the Hong Kong Research Grants Council. We are indebted to Professor C Hazlett and Professor M Hjelm for their advice and to Miss Rebecca Yau and Mr K K Man for their assistance during the preparation of this manuscript.

### REFERENCES

Alpert E and Feller ER (1978) Alpha-fetoprotein (AFP) in benign liver disease: evidence that normal liver regeneration does not induce AFP synthesis. *Gastroenterology* **74**: 856–858

Aoyagi Y, Suzuki Y, Isemuna M, Soga K, Ozaki T, Ichida T, Inoue K, Sasaki H and Ichida F (1984) Differential reactivity of alphafetoprotein with lectins and evaluation of its usefulness in the diagnosis of hepatocellular carcinoma. *Gann* **75**: 809–815

Aoyagi Y, Isemura M, Yosizawa Z, Suzuki Y, Sekine C, Ono T and Ichida F (1985) Fucosylation of serum-alpha-fetoprotein in patients with primary hepatocellular carcinoma. *Biochim Biophys Acta* **830**: 217–223

Aoyagi Y, Suzuki Y, Igarashi K, Saitoh A, Oguro M, Yokota T, Mori S, Suda T, Isemura M and Asakura H (1993) Carbohydrate structures of human alpha-fetoprotein patients with hepatocellular carcinoma: presence of fucosylated and non-fucosylated triantennary glycans. *Br J Cancer* **67**: 486–492

Bloomer JR, Waldmann TA, Mcintire KR and Klatskin G (1975) Alpha-fetoprotein in nonneoplastic hepatic disorders. *JAMA* **233**: 38–41

Buamah PK, Harris R, James DFW and Skillen AW (1986) Lentil lectin-reactive alphafetoprotein in the differential diagnosis of benign and malignant liver disease. *Clin Chem* **32**: 2083–2084

Burditt LJ, Johnson MM, Johnson PJ and Williams R (1994) Detection of hepatocellular carcinoma-specific alpha-fetoprotein by isoelectric focusing. *Cancer* **74**: 25–29

Colombo M, Franchis DR, Ninno ED, Sangiovanni A, Fazio CD, Tommasini M, Donato MF, Piva A, Carlo VD and Dioguardi N (1991) Hepatocellular carcinoma in Italian patients with cirrhosis. *N Eng J Med* **325** 675–680

Curley S, Izzo F, Gallipoli A, Bellis MD, Cremona F and Parrsi V (1995) Identification and screening of 416 patients with chronic hepatitis at high risk to develop hepatocellular carcinoma. *Ann Surg* **222**: 375–383

Du M-Q, Hutchinson WL, Johnson PJ and Williams R (1991) Differential alpha-fetoprotein lectin binding in hepatocellular carcinoma: diagnostic ultility at low serum levels. *Cancer* **67**: 476–480

Govindarajan S, Fong TL and Aschavai M (1987) Concanavalin A affinity of alpha-fetoprotein. *Am J Clin Pathol* **88**: 722–724

Ho S, Cheng P, Yuen J, Chan A, Leung N, Yeo W, Leung T, Lau WY, Li AKC and Johnson PJ (1996) Isoelectric focusing of alpha-fetoprotein in patients with hepatocellular carcinoma: frequency of specific banding patterns at non-diagnostic levels. *Br J Cancer* **73**: 985–989

Johnson PJ, Ho S, Cheng P, Chan A, Leung WT and Yuen J (1995) Germ cell tumours express a specific alpha-fetoprotein variant detectable by isoelectric focusing. *Cancer* **75**: 1663–1668

Johnson PJ, Portmann B and Williams R (1978) Alpha-fetoprotein concentration measured by radioimmunoassay in the diagnosing and excluding of hepatocellular carcinoma. *Br Med J* **2**: 661–663

Johnson PJ and Williams R (1980) Serum alpha-fetoprotein estimations and doubling time in hepatocellular carcinoma: influence of therapy and possible value in early detection. *J Natl Cancer Inst* **64**: 1329–1332

Johnson PJ and Williams R (1987) Cirrhosis and the aetiology of hepatocellular carcinoma. *J Hepatol* **4**: 140–147

Kew MC and Popper H (1984) Relationship between hepatocellular carcinoma and cirrhosis. *Sem Liver Dis* **4**: 136–146

Krusius T and Ruoslahti E (1982) Carbohydrate structure of the concanavalin A molecular variants of AFP. *J Biol Chem* **257**: 3453–3458

Lee HS, Chung YH and Kim CY (1991) Specificity of serum alpha-fetoprotein in HBsAg+ and HBsAg– patients in the diagnosis of hepatocellular carcinoma. *Hepatology* **14**: 68–72

Liaw Y-F, Tai D-I, Chu C-M Lin DY, Sheen IS, Chen JJ and Pao CC (1986) Early detection of hepatocellular carcinoma in patients with chronic type B hepatitis. A prospective study. *Gastroenterology* **90**: 263–267

Lok ASF and Lai CL (1989) α-fetoprotein monitoring in Chinese patients with chronic hepatitis B virus infection: role in early detection of hepatocellular carcinoma. *Hepatology* **9**: 110–115

Oka H, Kurioka N, Kim K, Kanno T, Tetsuo K, Mizoguchi Y and Kobayashi K (1990) Prospective study of early detection of hepatocellular carcinoma in patients with cirrhosis. *Hepatology* **12**: 680–687

Oka H, Tamori A, Kuroki T, Kobayashi K and Yamamoto S (1994) Prospective study of α-fetoprotein in cirrhotic patients monitored for development of hepatocellular carcinoma. *Hepatology* **19**: 61–66

Sato Y, Nakata K, Kato Y, Shima M, Ishi N, Koji T, Taketa K, Endo Y and Nagataki S (1993) Early recognition of hepatocellular carcinoma based on altered profiles of alpha-fetoprotein. *N Engl J Med* **328**: 1802–1806

Sawabu N and Hattori N (1987) Serological tumor markers in hepatocellular carcinoma. In *Neoplasms of the Liver*, Okuda K and Ishak KG (eds), pp. 227–238. Springer-Verlag: Tokyo

Taketa K, Izumi M and Ichikawa E (1983) Distinct molecular species of human alpha-fetoprotein due to differential affinities to lectins. *Ann N Y Acad Sci* **417**: 61–68

 © Cancer Research Campaign 1997